# Multi-Leakage Source Localization of Safety Valve Based on Uniform Circular AE Array and Improved MUSIC Algorithm

**DOI:** 10.3390/s23094515

**Published:** 2023-05-06

**Authors:** Jianjun Hou, Shuxun Li, Lingxia Yang

**Affiliations:** 1 School of Petrochemical Technology, Lanzhou University of Technology, Lanzhou 730050, China; 211080706002@lut.edu.cn; 2 Machinery Industry Pump Special Valve Engineering Research Center, Lanzhou 730050, China

**Keywords:** safety valve, acoustic emission, leak source location, uniform circular array, improved MUSIC algorithm

## Abstract

The safety valve is the core component of the pressure-relief protection device for pressure-bearing special equipment. When the safety valve leaks, the medium of the pressure vessel will be lost and wasted, which may cause safety accidents. With the aim to solve the problem of accurately locating the multiple leakage sources of safety valves, a localization method combining a uniform circular array acoustic emission detection and an improved multiple signal classification (MUSIC) algorithm is proposed. First, an improved wavelet threshold function denoising method is introduced to extract acoustic emission signals with high SNR, thereby reducing the rank of the covariance matrix, weakening the noise dispersion caused by eigenvalue reconstruction, avoiding signal and noise cross-confusion, and improving positioning accuracy. By introducing a windowed fast Fourier transform (FFT) frequency division processing link to obtain narrowband signal, the premise of using MUSIC positioning algorithm is established. In addition, a forward/backward spatial smoothing algorithm is introduced in the decoherence link to reduce co-channel interference, reduce the rank loss of the signal covariance matrix, and improve the positioning accuracy of the algorithm. The results show that when the working pressure is 0.70 MPa, 0.75 MPa, and 0.80 MPa, the deviation between the azimuth angle and elevation angle positioning results of each leakage source obtained by the improved MUSIC algorithm and the actual angle does not exceed 2°, and the relative error does not exceed 3.5%. Therefore, the improved MUSIC algorithm can accurately locate multiple leakage sources of the safety valve, and as the working pressure of the safety valve increases, the positioning accuracy of the improved MUSIC algorithm also increases accordingly.

## 1. Introduction

The safety valve is an automatic valve that uses the pressure of the medium itself to discharge a rated amount of fluid to prevent overpressure damage to pressure-bearing devices and equipment such as boilers, pressure vessels, or pressure pipelines due to pressure exceeding a predetermined safety value. Therefore, the normal operation of the equipment and the safety of personnel are guaranteed. However, the safety valve is prone to leakage, which will cause the loss and leakage of the medium and serious safety accidents [1,2,3]. Especially when the medium in the pressure equipment is toxic, highly corrosive, or a type of other dangerous medium, the leakage of the safety valve can easily cause major public safety accidents. At present, there have been many cases of accidents in which on-site staff have been poisoned due to a failure to discover and deal with the leakage source of the safety valve in time. The leakage of the safety valve is mainly caused by the damage of the sealing surface, and the damage of the sealing surface mainly has the following three reasons: scratches caused by steam erosion of the disc, erosion of corrosive medium, and erosion scratches of high-speed medium.

The traditional detection method of a safety valve requires the judgment of experienced technicians through listening for telling sounds or by removing the safety valve from the pipeline for off-line detection, which is both time-consuming and laborious, and the internal leakage of the safety valve cannot be grasped in time. Acoustic emission (AE) testing technology is a dynamic nondestructive testing method, which has the advantages of convenient testing, low cost, and does not require production to stop [4,5,6]. This technology can be used to monitor the leakage of the safety valve, and the leakage acoustic emission signal combined with the sound source location algorithm can be used to locate the leakage point of the safety valve so as to repair and replace the damaged safety valve in time, prevent the waste of media, and reduce the probability of accidents, resulting in an important research significance. At present, there is a lack of research on the leakage source location technology of large-diameter valves, and most of the leakage location research comes from the fields of oil and gas pipelines or pressure vessels. The most common leak location research method is based on the time difference of arrival (TDOA) principle. Some scholars have introduced the cross-correlation algorithm to further estimate the time delay on the basis of the original time difference of the arrival principle algorithm. The leak detection of structural pressure vessels or pipelines is not suitable for such irregular structures as safety valves [7,8,9]. The time reversal (TR) location method is also often used in the leak location of pressurized pipelines. Some scholars use signal processing technology to denoise the collected signals or use adaptive elimination to improve the positioning accuracy of the time reversal location algorithm. This method is suitable for linear sensor arrays and is not suitable for safety-valve-leak location [10,11]. The positioning method combining the cross-time spectrum (CTFS), hyperbolic positioning algorithm, and acoustic emission sensor array has been proposed to locate large-scale pressure and long-distance pipeline leakage, but the denoising processing of the collected signal is not considered, resulting in the positioning accuracy not reaching the range within 3% [12,13,14,15]. Some scholars have proposed that wavelet packet analysis and wavelet transform are used to extract features of leakage signals to ensure the location accuracy of the leakage source [16,17,18,19,20], but the location algorithm is not suitable for each leakage condition. The adaptability was not analyzed, and the localization algorithm was not improved. The structure of the safety valve is more complicated than that of the pressure vessel and pressure pipeline, so the adaptability of the positioning algorithm to the leakage condition needs to be considered. In addition to the localization method of the acoustic sensor array, some leak localization methods based on physical signals such as inlet and outlet pressure and flow have also been proposed for the leak localization of pressure pipelines [21,22,23]. However, the outlet of the safety valve is in an atmospheric pressure environment, so it is not suitable to use this method to locate the leakage of the safety valve.

In summary, the detection and localization of pipeline leaks has been extensively studied, but the field of safety valves has received limited attention. Most of the existing leak localization algorithms are based on the plane coordinate positioning of the same horizontal position of the acoustic emission sensor and the leak source, and there is no case of three-dimensional spatial angle positioning. Moreover, the sound source localization algorithm used in existing research ignores key factors such as signal denoising, bandwidth applicability, and co-frequency interference, resulting in poor leak localization accuracy.

Therefore, a multiple safety valve leakage source location method combining the improved MUSIC algorithm with uniform circular array (UCA) AE is proposed. In terms of improving the MUSIC algorithm, an improved wavelet threshold function denoising method is added to extract the acoustic emission signal with a high signal-to-noise ratio (SNR), thereby reducing the rank of the covariance matrix of the MUSIC algorithm and weakening the eigenvalue reconstruction method. Signal and noise cross-aliasing are avoided to disperse the noise. In terms of improving the wavelet threshold function, a threshold function that is continuous at the threshold is constructed by using the one-sided attenuation characteristic of the elliptic equation, and an adjustment factor is introduced to optimize the threshold function. The MUSIC algorithm is suitable for narrowband signals, but the leakage signal of the safety valve is a broadband signal. In addition, the frequency of leakage signals collected by different AE sensors is almost the same, and there is co-frequency interference, and the signal array will receive coherent signals in different directions, resulting in the loss of the rank of the signal covariance matrix in the MUSIC algorithm, which in turn leads to inaccurate positioning. Therefore, by introducing the window FFT frequency division processing link and the forward/backward spatial smoothing algorithm decoherence link, an improved MUSIC algorithm is constructed to obtain narrowband signals, reduce co-frequency interference, and improve positioning accuracy. In addition, this paper also studies the relationship between the positioning accuracy of the improved MUSIC algorithm and the working pressure.

## 2. Uniform Circular Array MUSIC Positioning Principle

In recent years, another type of elastic wave source that is not directly related to the fracture mechanism (such as fluid leakage, friction, impact, combustion, etc.) is called the secondary acoustic emission source. The acoustic emission signal of safety valve leakage belongs to the secondary acoustic emission source. It is the elastic wave excited by the pressure gas hitting the wall surface of the valve seat or the wall surface material of the valve outlet when the pressure gas leaks at the leakage point of the safety valve. It is a continuous type of acoustic emission signal.

**Figure 1:**
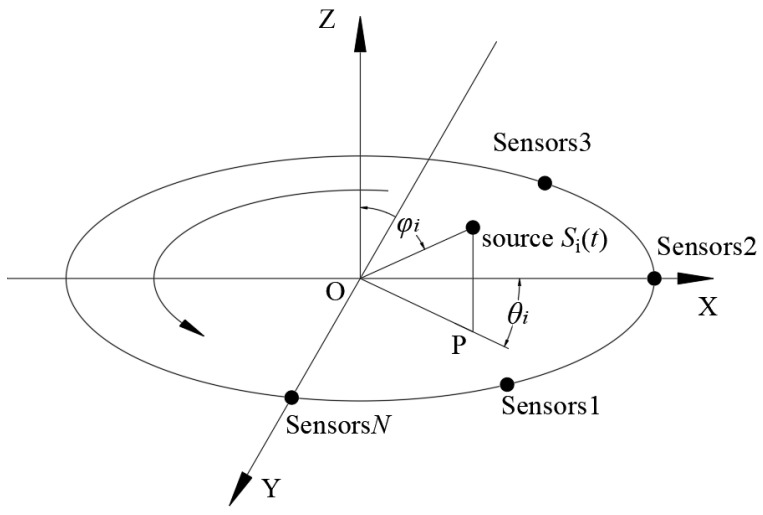
Uniform circular sensors array schematic model.

It can be seen from Figure 1 that *N*-sensors array elements are evenly distributed on a circle with a radius *R*. The AE sensor used in this study has a center frequency of 40 kHz. The interface is MS-KY, the frequency range is 16~60 kHz, the operating temperature is –20~130 °C, and the bottom is a piezoelectric ceramic surface. Assume that there are *M* far-field narrowband source signals *S*_1_(*t*), *S*_2_(*t*) …, *S_i_*(*t*), …*S_M_*(*t*) incident on the UCA, with the coordinate origin O as the reference point. The angle between the projection OP of any source signal *S*_*i*_(*t*) on the XOY-plane and the *X*-axis is the azimuth *θ_i_*, *θ_i_* ∈ [0°, 360°]. Connect any source signal to the center O to obtain O*S_i_*(*t*), and the angle between O*S_i_*(*t*) and the *Z*-axis is the pitch angle *φ_i_*, *φ_i_* ∈ [0°, 90°].

Suppose the positioning angle of the *i*-th source signal is (*θ_i_*, *φ_i_*), and the direction vector *α*(*θ_i_*, *φ_i_*) is the array response with angle (*θ_i_*, *φ_i_*). The expression of direction vector *α*(*θ_i_*, *φ_i_*) is as Formula (1):

(1) $$\alpha (\theta_{i},\varphi_{i})=\left[\begin{array}{c} e^{\left(j2\pi R\sin\varphi_{i}\cos(\theta_{i}-\eta_{0}\right)}{}^{/\lambda )}\\ e^{\left(j2\pi R\sin\varphi_{i}\cos(\theta_{i}-\eta_{1}\right)}{}^{/\lambda )}\\ \vdots \\ e^{\left(j2\pi R\sin\varphi_{i}\cos(\theta_{i}-\eta_{N-1}\right)}{}^{/\lambda )} \end{array}\right]$$

where *η_i_* is as Formula (2):

(2) $$\eta_{i}=2\pi n/N\;n=0,1,\ldots ,N-1$$

Then, the direction matrix *A* of *N* array elements can be expressed as:

*A* = [*α*(*θ*_1_*, φ*_1_), *α*(*θ*_2_, *φ*_2_)…, *α*(*θ_i_*, *φ_i_*),…*α*(*θ_M_*, *φ_M_*)] (3)

Thus, the received signal matrix *X*(*t*) of *N* array elements is obtained, as shown in Formula (4) [24].

(4) $$\begin{array}{l} X(t)=A*S\left(t\right)+N\left(t\right)={\left[x_{1}(t)x_{2}(t)\ldots x_{i}(t)\ldots x_{N}(t)\right]}^{T}\\ =\left[\begin{array}{cccc} e^{\left(j2\pi R\sin\varphi_{0}\cos(\theta_{0}-\eta_{0}\right)}{}^{/\lambda } & e^{\left(j2\pi R\sin\varphi_{1}\cos(\theta_{1}-\eta_{0}\right)}{}^{/\lambda } & \ldots & e^{\left(j2\pi R\sin\varphi_{M-1}\cos(\theta_{M-1}-\eta_{0}\right)}{}^{/\lambda }\\ e^{\left(j2\pi R\sin\varphi_{0}\cos(\theta_{0}-\eta_{1}\right)}{}^{/\lambda } & e^{\left(j2\pi R\sin\varphi_{1}\cos(\theta_{1}-\eta_{1}\right)}{}^{/\lambda } & \ldots & e^{\left(j2\pi R\sin\varphi_{M-1}\cos(\theta_{M-1}-\eta_{1}\right)}{}^{/\lambda }\\ & \vdots & & \\ e^{\left(j2\pi R\sin\varphi_{0}\cos(\theta_{0}-\eta_{N-1}\right)}{}^{/\lambda } & e^{\left(j2\pi R\sin\varphi_{1}\cos(\theta_{1}-\eta_{N-1}\right)}{}^{/\lambda } & \ldots & e^{\left(j2\pi R\sin\varphi_{M-1}\cos(\theta_{M-1}-\eta_{N-1}\right)}{}^{/\lambda } \end{array}\right]\cdot \left[\begin{array}{l} s_{1}(t)\\ s_{2}(t)\\ \ldots \\ s_{M}(t) \end{array}\right]+\left[\begin{array}{l} n_{1}(t)\\ n_{2}(t)\\ \ldots \\ n_{N}(t) \end{array}\right] \end{array}$$

In the formula, *S*(*t*) is the incident array signal; *N*(*t*) is the noise; *R* is the radius of the array element; and λ is the signal wavelength.

Based on the circular array receiving model, the mode space transformation technology is adopted, that is, the UCA direction vector *α*(*θ*, *φ*) is mapped to the beam space direction vector by using the beam transformation matrix *F_r_*, where the azimuth angle change in *β*(*θ*, *φ*) is similar to uniform linear array (ULA), which conforms to the Vandermonde matrix form, *β*(*θ*, *φ*) = F_r_*α(*θ*, *φ*), and the MUSIC algorithm can be used for positioning analysis [24]. Therefore, when the uniform circular array MUSIC algorithm is used to locate multiple leakage sources of safety valves, the pattern space transformation technology is first used to convert the received signal matrix information from the array element space to the beam space, and the beam space data vector is obtained:

(5) $$y(t)=F_{r}{}^{H}x(t)=F_{r}{}^{H}A*S(t)+F_{r}{}^{H}n(t)$$

The data covariance matrix is as Formula (6):

(6) $$M_{y}=E[y(t)y^{H}(t)]=F_{r}{}^{H}A*M_{X}*{\left(F_{r}{}^{H}A\right)}^{H}+\sigma^{2}I$$

where *M_X_* is the covariance matrix of the source signal; *y*(*t*)^**H**^ is the conjugate transpose matrix of the beam space data; *σ*^2^ is the noise power; and *I* is the identity matrix. When the eigenvalues of *M_y_* are decomposed, the eigenvectors corresponding to the large eigenvalues span into the beam space signal subspace, and the eigenvectors corresponding to the smaller eigenvalues span into the noise subspace, respectively:

(7) $$S=[u_{1},u_{2},\ldots ,u_{M}],\;G=[u_{M+1},u_{M+2},\ldots ,u_{N}]$$

where *S* is the signal subspace of the beam space and *G* is the noise subspace. The traditional MUSIC algorithm using space transformation technology is to replace the complex-valued eigendecomposition of the data covariance matrix *M_X_* in the array element space by decomposing the real-valued eigenvalues of the data covariance matrix *M_y_* in the beam space to obtain the signal subspace and the noise subspace.

In summary, the spatial–spectral function of the MUSIC algorithm under the UCA can be obtained as:

(8) $$P(\theta ,\varphi )=\frac{1}{\beta^{T}(\theta ,\varphi )GG^{T}\beta^{}(\theta ,\varphi )}$$

## 3. Improve the Construction of the MUSIC Algorithm

### 3.1. Wavelet Threshold Function Denoising to Extract High SNR Leakage Signal

Although the traditional MUSIC localization algorithm considers the noise, the covariance matrix of the noisy signal has a higher rank when the SNR is low. When the rank of the covariance matrix is high, the method of eigenvalue reconstruction will bring noise dispersion, which will make the signal and noise cross-confuse and require a high number of snapshots, causing the positioning effect of the MUSIC algorithm in practical engineering applications to be poor. In the actual safety valve leakage test environment, there are noises generated by mechanical equipment, such as compressors and piping systems. Therefore, before using the MUSIC algorithm, the signal collected by the AE sensors should be denoised to obtain a signal with a higher SNR to reduce the rank of the covariance matrix to prepare for the location of multiple leakage sources of the safety valve.

The wavelet threshold denoising method was proposed by Donoho et al. [25,26,27,28], including the hard threshold denoising method and soft threshold denoising method. This method has a small amount of calculation and is widely used. However, the method itself has defects; the wavelet hard threshold function is discontinuous, and oscillation may occur after noise reduction. Although the wavelet soft threshold has good continuity, there is a deviation between the processed wavelet coefficients and the real wavelet coefficients, and the error increases and the accuracy decreases when reconstructing the signal. Therefore, it is particularly important to choose an appropriate wavelet threshold function.

#### 3.1.1. Hierarchical Adaptive Threshold

In wavelet threshold denoising, it is necessary to set an appropriate threshold, which will act as a threshold to separate the noise from the useful signal in the signal. Commonly used thresholds include the unbiased likelihood estimation principle (rigrsure), the general threshold principle (sqtwlolg), the heuristic threshold principle (heursure), and the extreme-value threshold principle (minimax). Because of its wide application range, rigrsure is often used to solve practical engineering problems. Therefore, the rigrsure principle is used to obtain the threshold.

The coefficients of the three vectors decomposed by wavelet in Formula (4) correspond to the vectors w^*^_j,k_, *w_j,k_*, *q*, respectively, and the corresponding mathematical model is [29]:

(9) $$w^{*}{}_{j,k}=w_{j,k}+q$$

The square component vector of wavelet coefficient is:

(10) $$G=(w^{*}{}_{j,k}{)}^{2}=[g_{1},g_{2},\cdots ,g_{n}]$$

The thresholds are:

(11) λj=vjGminlog(j+1)

In the formula, *v_j_* is the noise standard deviation of different scales and *j* is the number of decomposition layers.

#### 3.1.2. Improved Wavelet Threshold Function

The AE signal of the safety valve leakage is submerged in the mechanical background noise, and the SNR is very low. Aiming at the shortcomings of soft and hard threshold functions, the new and improved wavelet threshold function has continuity in the wavelet domain, which overcomes the disadvantage of discontinuous points in the traditional wavelet threshold. To this end, an improved threshold function is proposed, as shown in Formula (12):

(12) $$w^{*}{}_{j,k}=\left(\begin{array}{ll} w_{j,k} & \left|w_{j,k}\right|>\left|M_{y}\right|\\ \mathrm{sgn}\left(w_{j,k}\right)\sqrt{\left(\lambda^{2}+2\lambda a\right)\left(1-\frac{{\left(\left|w_{j,k}\right|-\lambda -a\right)}^{2}}{a^{2}}\right)} & \left|M_{y}\right|\geq \left|w_{j,k}\right|\geq \lambda \\ 0 & \left|w_{j,k}\right|\leq \lambda \end{array}\right)$$

The axis *a* of the ellipse along the direction of the wavelet coefficient is the fine-tuning coefficient, and *b* is the other axis of the ellipse along the direction of the denoised wavelet coefficient. The ellipse is tangent to the function curve of the wavelet coefficient, and (*M_y_*, *M_x_*) is the point of tangency between the ellipse and the slope of the wavelet coefficient. When |*w_j,k_*| > |*M_x_*|, the hard threshold function is used for noise reduction; when |*M_y_*| > |*w_j,k_*| > *λ*, an elliptic function is proposed for noise reduction to avoid the discontinuity of the original hard threshold function at the |*w_j,k_*| = *λ* point.

(13) $$M(M_{x},M_{y})=\{\begin{array}{cc} \frac{b^{2}}{\lambda +a} & ,M_{x}\\ \frac{b^{2}}{\lambda +a}-2Ew_{j,k} & ,M_{y} \end{array}$$

**Figure 2:**
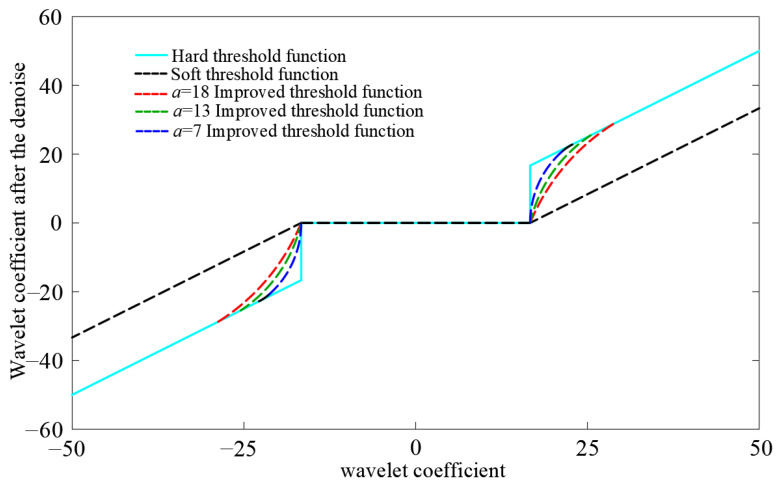
Comparison of different threshold function characteristics.

Figure 2 shows the soft and hard threshold functions and the improved threshold function with different coefficients for the major axis *a* of the ellipse. The new and improved wavelet threshold can adapt to various environments by choosing different values of the adjustment parameter *a*. The value of the adjustment parameter *a* is between 0 and 20. When *a* is smaller, the slope is larger, the curve is steeper, and the curve is closer to the hard threshold function; when *a* is larger, the slope of the curve is smaller and the curve is gentler.

To test the continuity of the improved threshold function, the continuity must firstly be verified at the positive semi-axis *w_j,k_* = *λ*:

(14) $$w_{j,k}^{*}\left((\lambda^{+}),a\right)=w_{j,k}^{*}\left((\lambda^{-}),a\right)=0$$

According to Equation (14), the improved threshold function is continuous at *w_j,k_* = *λ*. Similarly, the improved threshold function is continuous at *w_j,k_* = −*λ*. Therefore, the improved threshold function is continuous in the whole domain, which avoids the oscillation and Gibbs phenomenon in the signal reconstruction process.

### 3.2. Windowed FFT Frequency Division Processing

If the bandwidth *W_B_* of the signal to be analyzed and its center frequency *f_C_* satisfy Formula (15), it is regarded as a narrowband signal.

(15) $$W_{B}/f_{C}<0.1$$

The center frequency of the AE sensor to be used is 40 kHz, and the acquisition range of the AE acquisition instrument is 0~3 MHz, which belongs to broadband signal. However, the traditional MUSIC algorithm needs to satisfy the signal narrow-band assumption. Therefore, before locating the internal leakage source, the broadband signal after feature extraction is frequency-divided to obtain multiple narrow-band signals, and then the leakage source location research is carried out.

The essence of signal frequency division processing is to perform frequency domain conversion on the frame-divided signal based on windowing and framing. The window functions commonly used for framing and windowing processing includes rectangular window and Hamming window. The Hamming window with a wider main lobe and lower side lobe peak value is used to add window and framing to the numerical simulation internal leakage signal of a large-diameter pipeline ball valve. The expressions of Hamming window in the time domain and frequency domain are shown in Formulas (16) and (17), respectively [30,31]:

(16) $$\omega (n)=\left\{ \begin{array}{c} 0.54-0.46\cos[2\pi n/(N-1)],0\leq n\leq (N-1)\\ 0^{}{{}^{}}^{}{{}^{}}^{}{{}^{}}^{}{{}^{}}^{}{{}^{}}^{}{{}^{}}^{}{{}^{}}^{}{{}^{}}^{}{{}^{}}^{},n=else \end{array}\right.$$

(17) $$W(\omega )=0.54W_{R}(\omega )+0.23\left[W_{R}(\omega -\frac{2\pi }{N-1})+W_{R}(\omega +\frac{2\pi }{N-1})\right]$$

where *ω* is the angular frequency of the signal and *W_R_* is the assigned weight of the *R*-th source node. Based on windowing and framing, the FFT algorithm is used to divide the frequency of each frame signal to realize the narrowing of the leakage signal after improved wavelet threshold denoising, which provides the basis for subsequent positioning.

### 3.3. Backward and Forward Spatial Smoothing Algorithm for Decoherence

Because the frequency of endoleak signals collected by different AE sensors is the same, there is co-frequency interference, and the signal array will receive coherent signals in different directions, resulting in the lack of rank of the signal covariance matrix and inaccurate positioning. The leaky signal uses a forward/backward spatial smoothing algorithm, which is an effective decoherent method. Because the spatial smoothing algorithm is suitable for ULA, the UCA signal reception matrix in the array space is transformed into the ULA signal reception matrix in the beam space through the mode space transformation technology, and then the front and rear spatial smoothing processes are performed to decorrelate the signal.

**Figure 3:**
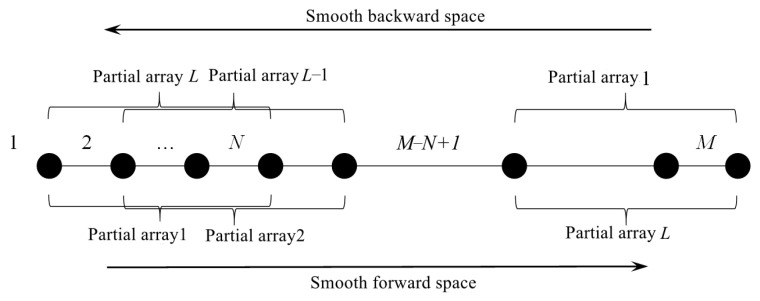
Schematic diagram of forward and backward spatial smoothing algorithm.

The basic principle of the spatial smoothing algorithm is to divide the receiving matrix of the array element into several overlapping subarrays. If the direction vectors of the subarrays are the same, the subarray data covariance matrices are added and averaged to replace the original covariance matrix. The schematic diagram of forward/backward spatial smoothing is shown in Figure 3. The forward spatial smoothing algorithm divides the *M*-element array into equidistant *L* subarrays, and each subarray contains *N* array elements. The backward spatial smoothing is the conjugate inversion of the forward spatial smoothing.

The output of the *l*-th forward subarray is defined as [32]:

(18) $$x_{l}^{f}(t)={\left[x_{l}(t),x_{l+1}(t),\cdot \cdot \cdot x_{l+N-1}(t)\right]}^{T}=A_{M}D^{l-1}s(t)+n_{l}(t),1\leq l\leq L$$

where *A*_M_ is an *N* × *K*-dimensional direction matrix, and its columns are *N*-dimensional steering vectors $a_{M}(\theta_{i})(i=1,2,\cdot \cdot \cdot ,K)$.

Therefore, the covariance matrix of the *l*-th forward subarray is:

(19) $$M_{l}^{f}=E[x_{l}^{f}(t)x_{l}^{f}{\left(t\right)}^{H}]=A_{M}D^{l-1}R_{S}{\left(D^{l-1}\right)}^{H}A_{M}^{H}+\sigma^{2}I$$

where *R_S_* is the source covariance matrix that remains as nonsingular so long as the sources are at most partially correlated and *D^l^*^−1^ is (*l* − 1)th power of the diagonal matrix. In this case, it can be determiend that the forward spatial smoothing covariance matrix of *L* subarrays is as in Formula (20):

(20) $$M_{f}=\frac{1}{L}\sum_{l-1}^{L}R_{l}^{f}$$

where $R_{l}^{f}$ is *l*-th forward spatial smoothing covariance matrix. By arranging Formula (20) backward, the backward spatial smoothing covariance matrix can be obtained:

(21) $$M_{b}=\frac{1}{L}\sum_{l-1}^{L}R_{l}^{b}$$

where $R_{l}^{b}$ is *l*-th backward spatial smoothing covariance matrix. Based on the conjugate flashback invariance of *M_f_* and *M_b_*, after converting the UCA-receiving matrix to ULA mode, the covariance matrix formed by processing the receiving matrix Formula (4) with forward and backward spatial smoothing algorithm is shown in Formula (22):

(22) $${\tilde{M}}_{y}=\frac{1}{2}(M_{f}+M_{b})$$

In the formula, *M_f_* represents the forward spatial smoothing covariance matrix and *M_b_* is the backward spatial smoothing covariance matrix.

### 3.4. Improved MUSIC Algorithm Spatial–Spectral Function Derivation

The eigenvalues of the covariance matrix ${\tilde{M}}_{y}$ decompose after the forward and backward smoothing of Formula (22), the larger *M* eigenvalues are then arranged in descending order *λ*_1_ > *λ*_2_ > … > *λ_M_*, and the *M* larger eigenvalues correspond to the eigenvector, as stated by Zhang. In the signal subspace of the beam space, the eigenvectors corresponding to the smaller *N* m eigenvalues form the noise subspace, and the signal subspace and the noise subspace are shown in Formula (23):

(23) $$\tilde{S}=[\tilde{u_{1}},\tilde{u_{2}},\ldots ,\tilde{u_{M}}],\tilde{G}=[{\tilde{u}}_{{}_{M+1}},{\tilde{u}}_{{}_{M+2}},\ldots ,\tilde{u_{N}}]$$

From this, the spatial spectrum function of the improved MUSIC algorithm can be obtained as:

(24) $$P(\theta ,\varphi )=\frac{1}{\tilde{\beta^{T}}(\theta ,\varphi )\tilde{G^{}}\tilde{G^{T}}\tilde{\beta^{}}(\theta ,\varphi )}$$

In the formula, $\tilde{\beta^{}}(\theta ,\varphi )$ is the subarray direction vector obtained by the forward and backward space smoothing algorithm after the pattern space transformation. Through Formula (24), the peak search is carried out in the two-dimensional space of azimuth angle *θ* and elevation angle *φ*, and the corresponding angles of *M* maximum points are obtained to determine the location of the safety valve leakage source.

## 4. Safety Valve Leak Test and Noise Reduction of Leakage Signals

### 4.1. Safety Valve Basic Parameters

**Figure 4:**
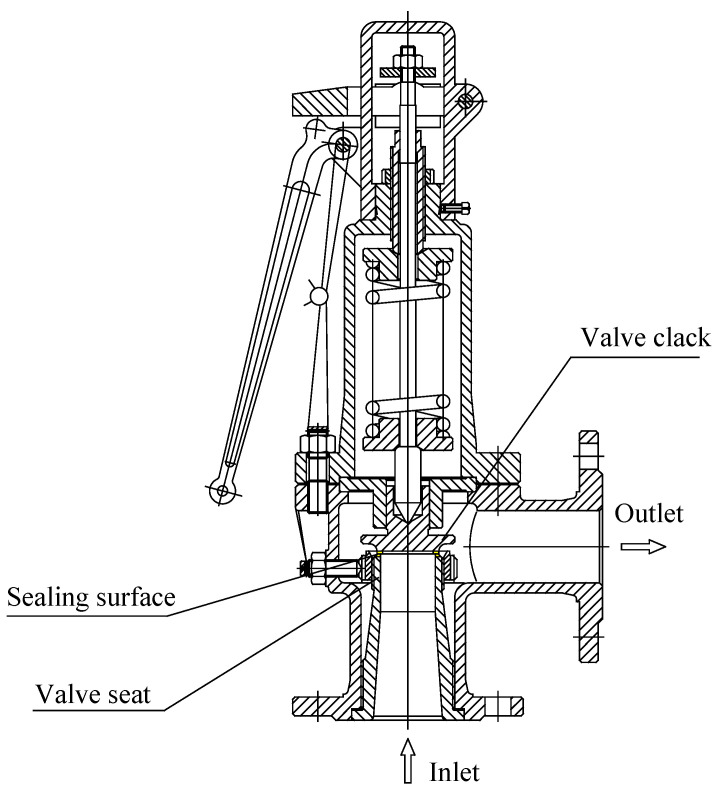
Spring-loaded safety valve.

The structure of the safety valve used in the AE leak test is shown in Figure 4. The position where the valve seat contacts the disc is the sealing surface of the safety valve. When the sealing surface of the safety valve is damaged, the pressure gas will be ejected from the damaged sealing surface, causing the safety valve to leak. Materials of the main parts of the valve are shown in Table 1. The performance parameters of the safety valve are shown in Table 2.

### 4.2. Safety Valve Leakage Signal Acquisition

**Figure 5:**
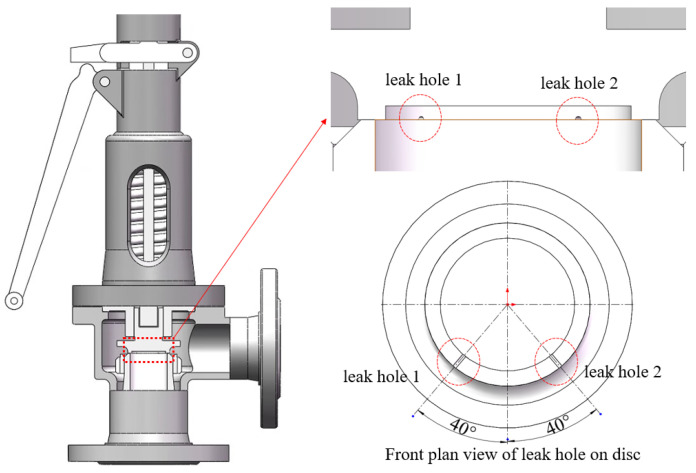
Schematic diagram of safety valve leakage position.

**Figure 6:**
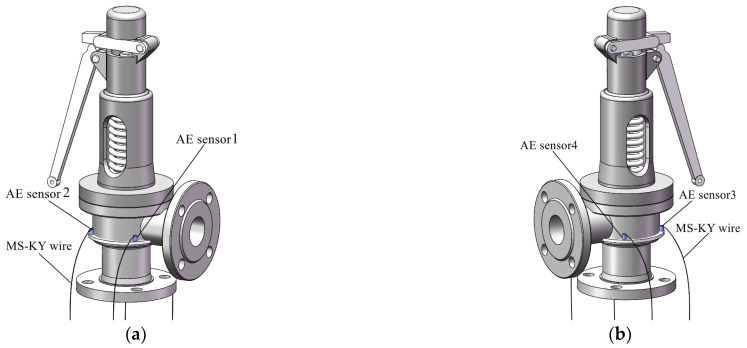
Three-dimensional schematic diagram of safety valve sensors’ position. (**a**) front view; (**b**) rear view.

**Figure 7:**
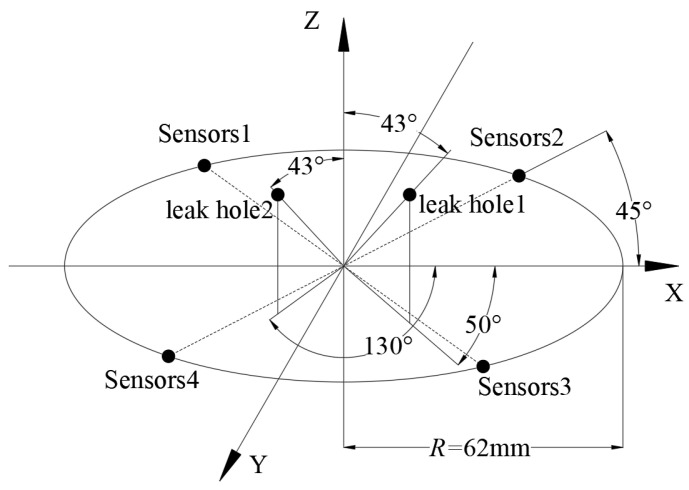
Coordinates of leak source and AE sensors.

In order to verify the location accuracy of the improved MUSIC algorithm, two φ1mm semicircular leakage holes are opened on the safety valve disc. The center line of the holes is 40° to the center of the safety valve, as shown in Figure 5. On the circumference of the valve body and on the lower side of the leakage hole, four AE sensors are evenly arranged at intervals of 90° to form a circular array, as shown in Figure 6. The UCA sensors array is fixed as close to the sealing surface as possible to reduce signal attenuation. Taking the plane where the sensors circular array is located as the XOY-plane, and taking the central axis of the safety valve as the *Z*-axis, a coordinate system is constructed, as shown in Figure 7. It can be seen from Figure 7 that the spatial angular coordinates of the four AE sensors are (45°, 0°), (135°, 0°), (225°, 0°), and (315°, 0°), respectively. The spatial angular coordinates of the leak hole center are (50°, 43°) and (130°, 43°).

**Figure 8:**
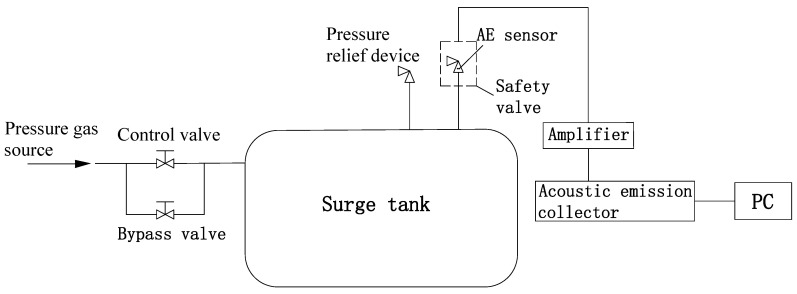
Flow chart of safety valve leakage test system.

**Figure 9:**
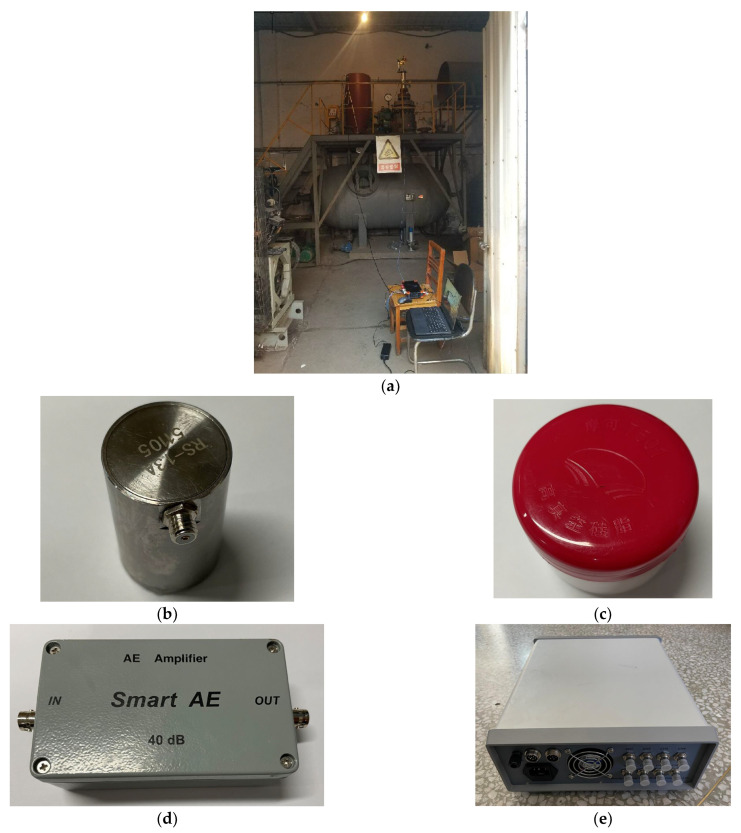
Safety valve leakage detection system and instruments. (**a**) The system of safety valve leakage testing; (**b**) RS-13A AE sensor; (**c**) coupling agent; (**d**) signal amplifier; (**e**) AE acquisition instrument.

Figure 8 is a flow chart of the safety valve leakage test system and Figure 9a shows the test system. The compressor provides the pressure gas source and the pressure gas source reaches the surge tank through the control valve and the bypass valve. In the case of a safety valve leak, pressurized gas flows through the safety valve leak hole located in the surge tank, resulting in an AE signal. The acoustic emission signals of the safety valve are tested at working pressures of 0.70 MPa, 0.75 MPa, and 0.80 MPa, respectively.

The acoustic emission sensor, coupling agent, amplifier, and signal acquisition instrument are shown in Figure 9b–e. The acoustic emission sensor model is RS-13A, the interface is MS-KY, the center frequency is 40 kHz, the frequency range is 16~60 kHz, the operating temperature is −20~130 °C, and the bottom is a piezoelectric ceramic surface. The acoustic emission acquisition instrument has four channels, the acquisition frequency is 3MHz, the input signal range is ±10V, the channel input impedance is 50 ohms, and the operating temperature range is 10~50 °C. The amplification gain of the signal amplifier is 100 times, the maximum output voltage is ±10V, the interface type is BNC interface, and the operating temperature range is −20~60 °C. The couplant is a special silicone grease. It has good chemical stability, strong material adaptability, an applicable temperature range of −50~+220 °C, and has the function of lubrication.

**Figure 10:**
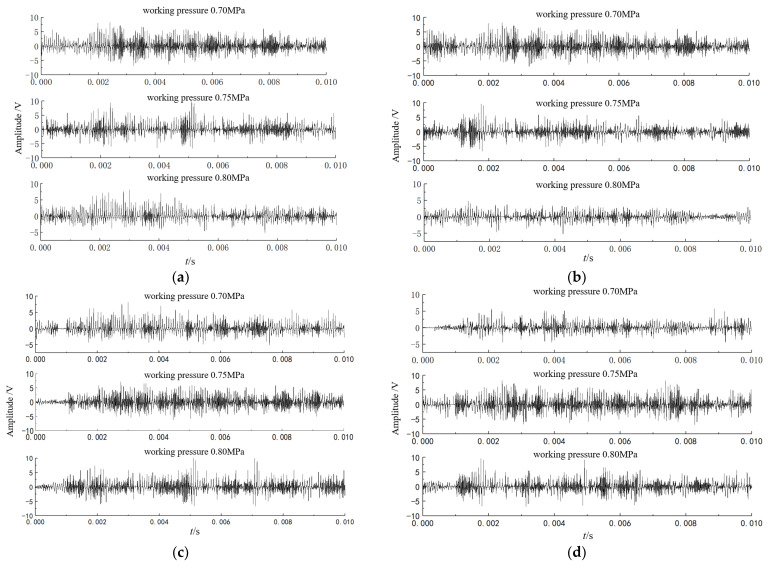
Safety valve leakage time domain signal. (**a**) Time domain signal from sensor 1; (**b**) time domain signal from sensor 2; (**c**) time domain signal from sensor 3; (**d**) time domain signal from sensor 4.

**Figure 11:**
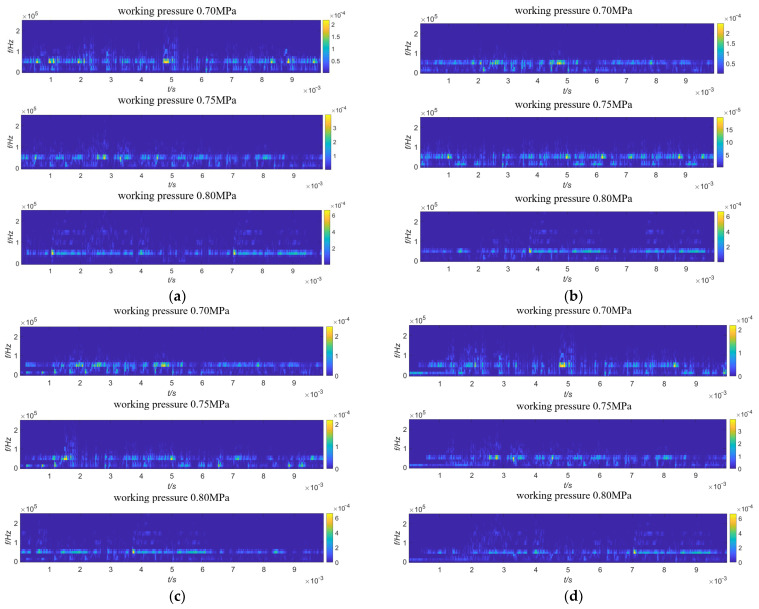
Wavelet transform diagram of leakage signal of safety valve. (**a**) Wavelet transform diagram of sensor 1; (**b**) wavelet transform diagram of sensor 2; (**c**) wavelet transform diagram of sensor 3; (**d**) wavelet transform diagram of sensor 4.

The AE time domain signals collected by AE sensors 1 to 4 under the gas source pressures of 0.70 MPa, 0.75 MPa, and 0.80 MPa are shown in Figure 10. Since the energy after wavelet transform is mainly distributed within 200 kHz, in order to express the time-frequency distribution of the signal more clearly, a partially enlarged view of wavelet transform from 0 to 200 kHz is taken, as shown in Figure 11. It can be seen from Figure 11 that the collected original AE signal is doped with a large number of low-frequency environmental noise signals, so it is necessary to denoise the signal before positioning to improve the SNR.

### 4.3. Improved Wavelet Threshold Function Denoising

The *SNR* and root mean square error (*RMSE*) after signal noise reduction are used as the evaluation criteria for the noise reduction effect. The larger the *SNR*, the smaller the *RMSE* value and the better the noise reduction effect. The calculation formulas of *SNR* and *RSME* are [33]:

(25) $$SNR=10\mathrm{lg}\left[\frac{\sum_{t=1}^{n}x^{2}(t)}{\sum_{t=1}^{n}{\left[x(t)-q(t)\right]}^{2}}\right]$$

(26) $$RSME=\sqrt{\frac{1}{n}\sum_{1}^{n}{\left[x(t)-q(t)\right]}^{2}}$$

where *x*(*t*) is the noise-containing signal; *q*(*t*) is the signal-after-noise reduction, and *n* is the length of the noise-containing signal. Taking the signal collected by AE sensor 1 under the working pressure of 0.70 MPa as an example, the adjustment factor *a* = 18 is selected to study the influence of different decomposition layers on the noise reduction effect of the tested signal, as shown in Table 3. The properties of different wavelet basis functions are shown in Table 4. Haar, Daubechies (*N*), and Symlets (*N*) have good characteristics and small vanishing moments, so the influence of these three wavelet basis functions on the noise reduction effect is studied, as shown in Table 5. When the number of decomposition layers is 3 and the wavelet basis function is db6, the denoised signal has a higher *SNR* and smaller *RMSE*.

**Figure 12:**
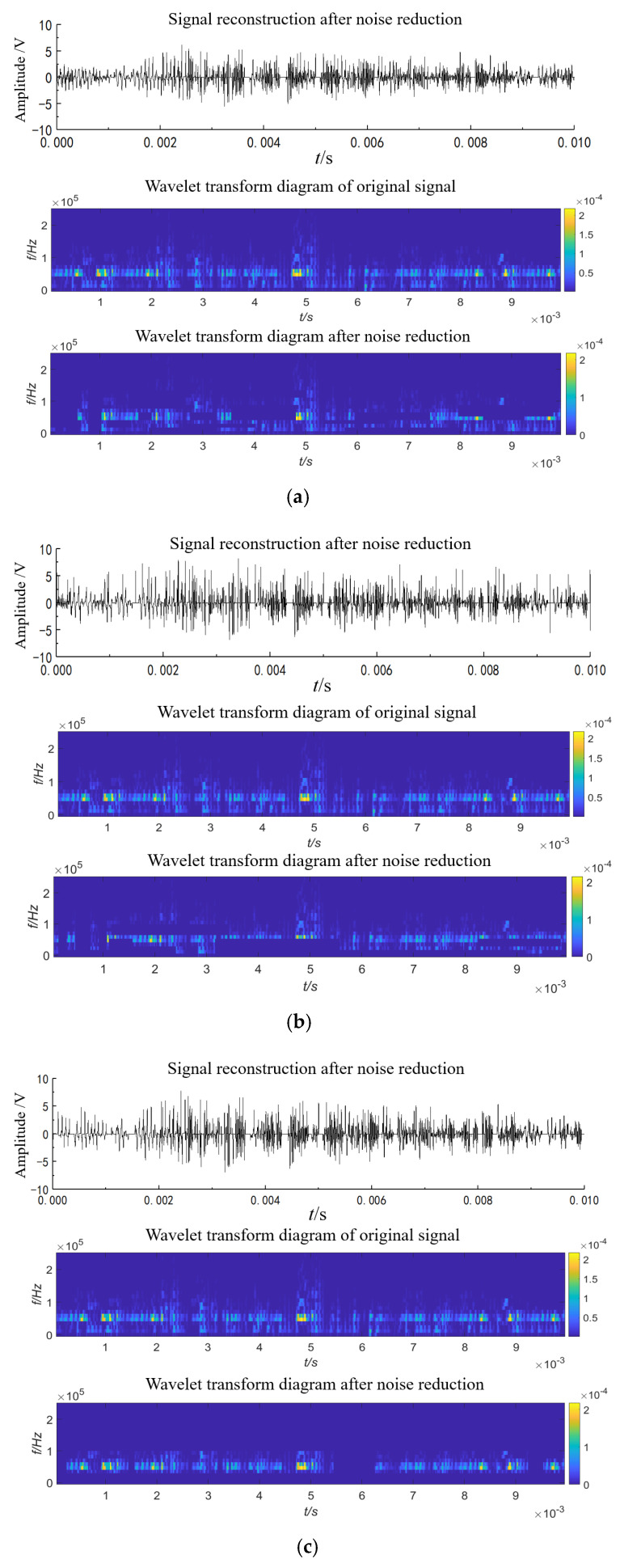
Threshold denoising. (**a**) Soft-threshold denoising; (**b**) hard-threshold denoising; (**c**) improved wavelet threshold denoising.

The three-layer decomposition, Rigrsure threshold, and db6 wavelet basis function are selected. The soft threshold, hard threshold, and improved threshold functions are used to reduce the noise of the gas source pressure 0.70 MPa signal collected by sensor 1. The denoised signal is shown in Figure 12. Both soft-threshold and hard-threshold denoising retain some low-frequency environmental noise and remove some high-frequency leakage signal noise, and the noise reduction effect is poor. The improved wavelet threshold function can eliminate environmental noises below 20 kHz, such as compressors and pipeline vibrations. The final signal is distributed around the leakage center frequency of 40 kHz, and the noise reduction effect is improved.

**Figure 13:**
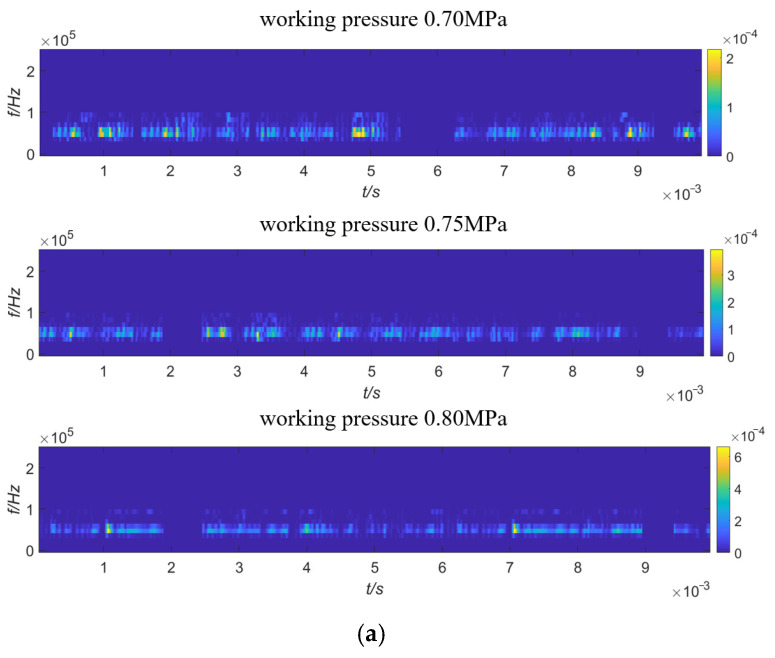

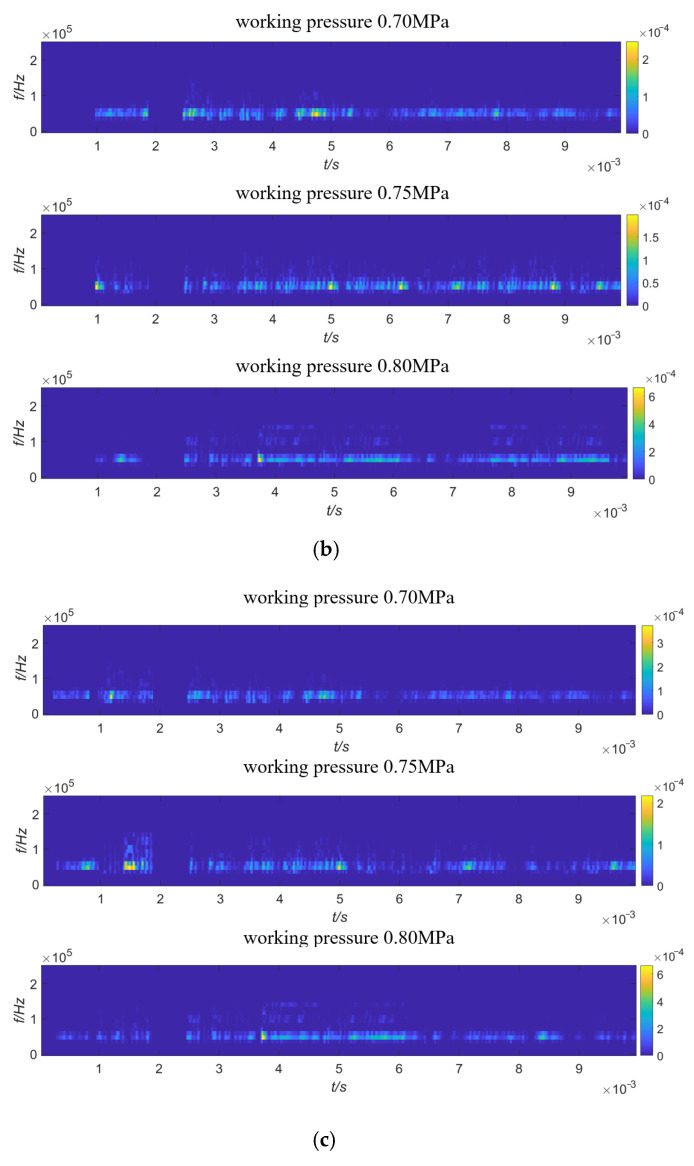

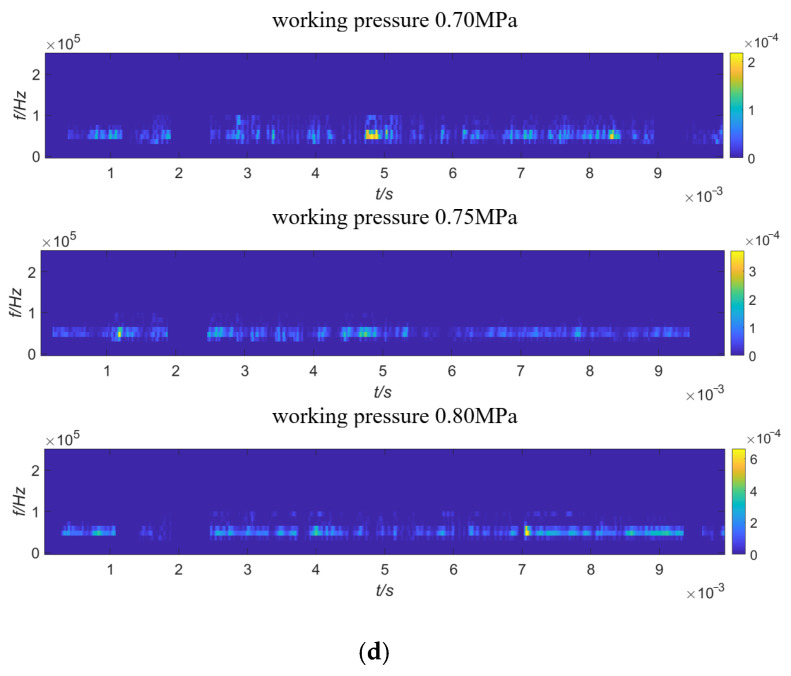
Wavelet transform diagram after improved wavelet threshold denoising. (**a**) Sensor no. 1; (**b**) sensor no. 2; (**c**) sensor no. 3; (**d**) sensor no. 4.

The *SNR* and *RMSE* of the denoised signal with soft threshold, hard threshold, and improved threshold functions are shown in Table 6. The signal-to-noise ratio of the improved threshold function denoising is 18.8 times that of the soft threshold function and 7.69 times that of the hard threshold function. The improved wavelet threshold function is used to denoise the signals of all working conditions collected by sensors 1–4 and perform wavelet transformation on the denoised signals, as shown in Figure 13. The signal energy after noise reduction is mainly concentrated in the center frequency of the safety valve leakage signal; around 40 kHz, the noise reduction effect is good and the interference of low-frequency signals, such as compressors and pipeline vibrations, on the positioning accuracy of the improved MUSIC algorithm is reduced.

## 5. Results and Discussion

### 5.1. Results

**Figure 14:**
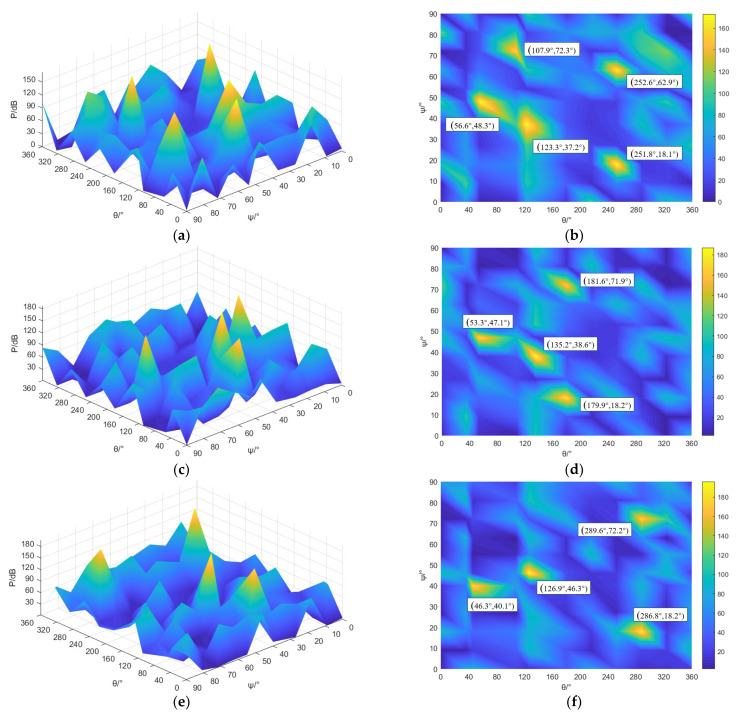
Localization results of multiple leakage sources of safety valves based on traditional MUSIC algorithm. (**a**) 0.70 MPa space angle spectrum; (**b**) 0.70 MPa angle spectrum, top view; (**c**) 0.75 MPa space angle spectrum; (**d**) 0.75 MPa angle spectrum, top view; (**e**) 0.80 MPa space angle spectrum; (**f**) 0.80 MPa angle spectrum, top view.

The traditional MUSIC algorithm and the improved MUSIC algorithm are programmed based on MATLAB, respectively, and the space angle spectrum of the traditional MUSIC algorithm and the improved MUSIC algorithm under the working pressure of 0.70 MPa, 0.75 MPa, and 0.80 Mpa are obtained through numerical calculation. It can be seen from Figure 14 that the number of peak coordinates of the leakage position of the safety valve with working pressures of 0.70 MPa, 0.75 MPa, and 0.80 Mpa based on the traditional MUSIC algorithm exceeds 2. Among them, there are five peak coordinates in the space angle spectrum of the MUSIC algorithm with a gas source pressure of 0.70 MPa, and it is difficult to directly determine the true peak representing the position of the leak hole and the false peak that interferes with the judgment. The coordinates corresponding to the two peaks that are closer to the actual leak center coordinates are (56.6°, 48.3°) and (123.3°, 37.2°), respectively, but these two coordinates are different from the actual leak source center position coordinates (50°, 43°) and (130°, 43°), which are quite different. The coordinates corresponding to the remaining three peaks are far away from the real leakage center coordinates, and these three peaks are false peaks. There are more than two peak coordinates in the traditional MUSIC algorithm space angle spectrum with gas source pressures of 0.75 MPa and 0.80 MPa. It is difficult to directly determine the true peak representing the position of the leak hole and the false peak that interferes with the judgment. Under the condition of an air source pressure of 0.75 MPa, the coordinates corresponding to the two peaks which are close to the coordinates of the actual leakage center are (53.3°, 47.1°) and (135.2°, 38.6°); under the condition of an air source pressure of 0.80 MPa, the coordinates corresponding to the two peaks that are closer to the actual leakage center coordinates are (46.3°, 40.1°) and (126.9°, 46.3°), respectively. However, these two coordinates are quite different from the coordinates (50°, 43°) and (130°, 43°) of the actual leak source center position. The traditional MUSIC algorithm of the three working conditions has a relatively high spatial angle spectrum function base, which leads to poor recognition of the positioning image.

**Figure 15:**
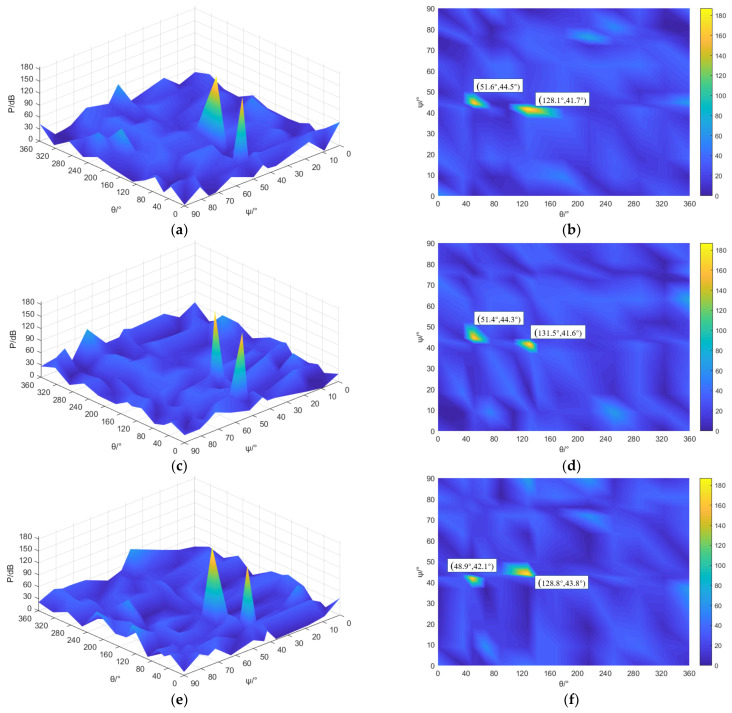
Localization results of multiple leakage sources of safety valves based on improved MUSIC algorithm. (**a**) 0.70 MPa space angle spectrum; (**b**) 0.70 MPa angle spectrum, top view; (**c**) 0.75 MPa space angle spectrum; (**d**) 0.75 MPa angle spectrum, top view; (**e**) 0.80 MPa space angle spectrum; (**f**) 0.80 MPa angle spectrum, top view.

It can be seen from Figure 15 that by improving the MUSIC positioning algorithm, the number of spatial angle spectrum peaks in each working condition is two, the spectrum base is low, and the positioning image recognition degree is better. When the gas source pressure is 0.70 MPa, 0.75 MPa, or 0.80 MPa, the peak coordinates of the improved MUSIC spatial angle spectrum are, respectively, located at (51.6°, 44.5°), (128.1°, 41.7°); (51.4°, 44.3°), (131.5°, 41.6°); (48.9°, 42.1°), (128.8°, 43.8°). Compared with the actual leakage area center coordinates (50°, 43°), (130°, 43°), it can be concluded that the location coordinates of the leak source based on the improved MUSIC algorithm are near the center of the leak, and the positioning deviations of the azimuth and elevation angles are not more than 2°, so the improved MUSIC algorithm has a good location effect. Table 7 and Table 8 show the positioning coordinates of leakage hole 1 and leakage hole 2 in multiple tests under different pressure conditions of the improved MUSIC algorithm.

The positioning effect of the leakage source of the safety valve under each working pressure is judged by the relative error of the pitch angle and the azimuth angle. The calculation formulas of the relative error of the pitch angle and the azimuth angle are shown in Formulas (27) and (28):

(27) $$RE_{1}=\frac{\frac{1}{Z}\sum_{i=1}^{Z}\varphi_{i}}{\varphi_{0}}$$

(28) $$RE_{2}=\frac{\frac{1}{Z}\sum_{i=1}^{Z}\theta_{i}}{\theta_{0}}$$

In the formula, *Z* represents the number of experiments; *θ_i_* and *φ_i_* represent the azimuth and elevation angles of the source signal in the *i*-th experiment, respectively. *θ*_0_ and *φ*_0_ represent the true azimuth and elevation angles of the source signal, respectively. The location coordinates of the three tests of the two leakage holes substituted with working pressures of 0.70 MPa, 0.75 MPa, and 0.80 Mpa and the coordinates of the leakage center (50°, 43°), (130°, 43°) into the root mean square error formula, respectively, and the relative errors of the improved MUSIC algorithm in azimuth and elevation angles obtained under different pressures are shown in Table 9.

### 5.2. Discussion

The positioning deviations of the pitch angle φ and the azimuth angle θ of the two leakage source coordinates of the safety valve based on the improved MUSIC algorithm are both less than 2°, and the relative errors are less than 3.5%. Therefore, combining the circular AE sensors array testing technology and the improved MUSIC algorithm can accurately locate the multiple leakage sources of the safety valve. When the working pressure is 0.70 MPa, 0.75 MPa, and 0.80 MPa, based on the improved MUSIC algorithm, the relative positioning errors of the azimuth angle θ of the safety valve leakage hole 1 are 3.2%, 2.5%, and 2.1%, respectively, and the relative positioning errors of the pitch angle φ are 3.3%, 2.9%, and 2.2%, respectively. Based on the improved MUSIC algorithm, the relative positioning errors of the azimuth angle θ of the safety valve leakage hole 2 are 1.4%, 1.2%, and 0.9%, respectively, and the relative positioning errors of the pitch angle φ are 2.9%, 2.8%, and 1.9%, respectively. As the working pressure increases, the positioning accuracy of the MUSIC algorithm also increases accordingly.

This research is aimed at the leakage location research of the safety valve whose medium is gas, and then it will attempt to apply this leakage location method to the leakage location research of the safety valve whose medium is liquid. Whether this method of leak location can be generalized to liquid safety valves will be judged. The location accuracy of the leakage source of the two media safety valves based on the improved MUSIC algorithm will be compared, and the advantages and disadvantages of using this technology to test the leakage of various media will be analyzed.

## 6. Conclusions

Combined with uniform circular array AE and improved MUSIC algorithm, the location of multiple leakage sources of safety valves is studied, and the following conclusions are obtained.

The proposed improved wavelet threshold denoising function can effectively eliminate the Gibbs phenomenon in the signal reconstruction process, and it has a variable adjustment coefficient *a*, which allows the function to have an adaptive shrinkage adjusted according to the working conditions. The results show that the leakage signal of the safety valve after denoising by the improved wavelet threshold function has a high signal-to-noise ratio and can be used for the positioning of the positioning algorithm.

By substituting the denoised signal of the improved wavelet threshold function into the improved MUSIC algorithm with the FFT frequency division processing link and the forward and backward spatial smoothing algorithm decoherence link, the results show that the improved MUSIC algorithm can remove the false peaks of the spatial angle spectrum and reduce the spectrum base to improve the image recognition ability of the multiple leakage sources of safety valves.

Based on the improved MUSIC algorithm, the positioning deviations of the pitch angle φ and azimuth θ of the two leakage source coordinates of the safety valve are both less than 2°, and the relative error is less than 3.5%. Therefore, combining the circular AE sensors array detection technology and the improved MUSIC algorithm can accurately locate the multiple leakage sources of safety valves.

When the working pressure is 0.70 MPa, 0.75 MPa, or 0.80 MPa, from the positioning results of the improved MUSIC algorithm, it can be seen that as the working pressure increases, the positioning accuracy of the MUSIC algorithm also increases accordingly.

## Figures and Tables

**Table 1:** Materials of main parts of the valve.

Name	Material
Valve body	WCB
Seat	2Cr13
Disc	2Cr13
Spring	50CrVA

**Table 2:** Spring-loaded safety valve parameters.

Name	Parameter
PN/MPa	1.6
DN/mm	65
Discharge Pressure/MPa	≥0.82
Back Pressure/MPa	≥0.65
Temperature/°C	≤350 °C

**Table 3:** Effect of different decomposition layers on noise reduction.

Layers	Performance Parameters	Improved Threshold
1	*SNR* *RSME*	2.1658 5.0367
2	*SNR* *RSME*	3.2584 3.4265
3	*SNR* *RSME*	3.6187 3.2316
4	*SNR* *RSME*	3.1623 3.6891
5	*SNR* *RSME*	2.5894 4.6231

**Table 4:** Properties of common wavelet basis functions.

Wavelet Basis Function	Tight Support	Orthogonality	Symmetry	Vanishing Moment
Meyer	No	No	Yes	No
Haar	Yes	Yes	Yes	1
Daubechies (*N*)	Yes	Yes	Yes	*N*
Symlets (*N*)	Yes	Yes	Yes	*N*
Coiflets (*N*)	Yes	Yes	Yes	2*N*

**Table 5:** Influence of different wavelet basis functions on noise reduction.

Basis Function		Improved Threshold
Haar	*SNR* *RSME*	2.9853 4.2652
sym6	*SNR* *RSME*	3.1367 3.5912
db6	*SNR* *RSME*	3.6187 3.2316

**Table 6:** Comparison of *SNR* and *RSME* after different threshold function noise reduction.

Threshold Function	Evaluation Indicators	
	*SNR*/dB	*RMSE*/dB
Soft Threshold	0.1918	6.0163
Hard Threshold	0.4702	4.6079
Improvement Threshold	3.6187	3.2316

**Table 7:** Location coordinates of leakage hole 1 under different pressures.

Pressure/MPa	First Test	Second Test	Third Test
0.70	(51.6°, 44.5°)	(51.7°, 44.3°)	(51.5°, 44.5°)
0.75	(51.4°, 44.3°)	(51.2°, 44.3°)	(51.2°, 44.2°)
0.80	(48.9°, 42.1°)	(49.0°, 42.0°)	(49.0°, 42.1°)

**Table 8:** Location coordinates of leakage hole 2 under different pressures.

Pressure/MPa	First Test	Second Test	Third Test
0.70	(128.1°, 41.7°)	(128.3°, 41.6°)	(128.2°, 41.7°)
0.75	(131.5°, 41.6°)	(131.6°, 41.9°)	(131.5°, 41.8°)
0.80	(128.8°, 43.8°)	(128.9°, 43.7°)	(128.8°, 43.9°)

**Table 9:** Relative errors of azimuth angle and pitch angle under different pressures.

Pressure/MPa	Leak Hole 1		Leak Hole 2	
	*θ*	*φ*	*θ*	*φ*
0.70	3.2%	3.3%	1.4%	2.9%
0.75	2.5%	2.9%	1.2%	2.8%
0.80	2.1%	2.2%	0.9%	1.9%

## Data Availability

Not applicable.
